# How are gender inequalities facing India’s one million ASHAs being addressed? Policy origins and adaptations for the world’s largest all-female community health worker programme

**DOI:** 10.1186/s12960-018-0338-0

**Published:** 2019-01-08

**Authors:** R. Ved, K. Scott, G. Gupta, O. Ummer, S. Singh, A. Srivastava, A. S. George

**Affiliations:** 1 National Health Systems Resource Centre, National Institute of Health & Family Welfare Campus, Baba Gangnath Marg, Munrika, New Delhi, Delhi 110067 India; 2 Independent researcher, Bangalore, India; 3 Johns Hopkins School of Public Health, 615 N Wolfe Street, Baltimore, 21205 Maryland USA; 4 School of Public Health, University of the Western Cape, Robert Sobukwe Rd, Bellville, Cape Town, 7535 South Africa

**Keywords:** Gender, Community health workers, India, Policy analysis, Human resources for health

## Abstract

**Background:**

India’s accredited social health activist (ASHA) programme consists of almost one million female community health workers (CHWs). Launched in 2005, there is now an ASHA in almost every village and across many urban centres who support health system linkages and provide basic health education and care. This paper examines how the programme is seeking to address gender inequalities facing ASHAs, from the programme's policy origins to recent adaptations.

**Methods:**

We reviewed all publically available government documents (*n* = 96) as well as published academic literature (*n* = 122) on the ASHA programme. We also drew from the embedded knowledge of this paper’s government-affiliated co-authors, triangulated with key informant interviews (*n* = 12). Data were analysed thematically through a gender lens.

**Results:**

Given that the initial impetus for the ASHA programme was to address reproductive and child health issues, policymakers viewed volunteer female health workers embedded in communities as best positioned to engage with beneficiaries. From these instrumentalist origins, where the programme was designed to meet health system demands, policy evolved to consider how the health system could better support ASHAs. Policy reforms included an increase in the number and regularity of incentivized tasks, social security measures, and government scholarships for higher education. Residential trainings were initiated to build empowering knowledge and facilitate ASHA solidarity. ASHAs were designated as secretaries of their village health committees, encouraging them to move beyond an all-female sphere and increasing their role in accountability initiatives. Measures to address gender based violence were also recently recommended. Despite these well-intended reforms and the positive gains realized, ongoing tensions and challenges related to their gendered social and employment status remain, requiring continued policy attention and adaptation.

**Conclusions:**

Gender trade offs and complexities are inherent to sustaining CHW programmes at scale within challenging contexts of patriarchal norms, health system hierarchies, federal governance structures, and evolving aspirations, capacities, and demands from female CHWs. Although still grappling with significant gender inequalities, policy adaptations have increased ASHAs’ access to income, knowledge, career progression, community leadership, and safety. Nonetheless, these transformative gains do not mark linear progress, but rather continued adaptations.

## Background

The last decade has seen a resurgence of interest in national community health worker (CHW) programmes [1]. CHWs are paid or volunteer community members without professional degrees who are given basic training and work to improve health among their peers. CHWs have been positioned as an important element in partly overcoming the global health workforce shortage, thus enabling countries to achieve universal health coverage under Sustainable Development Goal 3 [2].

Within the recent flurry of attention towards CHWs, there has been little discussion of the gender considerations of these programmes. Yet every decision in designing, implementing and adapting CHW programmes has gendered implications: from deciding whose health to prioritize, which community members are selected with implications for livelihoods, safety, and job security, to (re)constructing gendered norms of caregiving and decision-making in families, communities, and healthcare systems. Although no comprehensive gender disaggregated data exists for CHW programmes, several countries have CHW programmes that are all-female by design. This is the case of the Lady Health Workers in Pakistan, the Women’s Development Army in Ethiopia, and India’s nearly one million-worker strong accredited social health activist (ASHA) programme (Table 1) [3].

**Table 1:** Background on the ASHA programme

India has a long history of CHW programmes in both the governmental and non-governmental sectors, beginning before the country gained independence in 1947 [17]. Government commissions on health, such as the Bhore Committee in 1946 [61] and Srivastava Committee in 1975 [62], laid out frameworks for Indian primary health care that included training and supporting local people to serve as outreach workers, first aid providers, health educators, and health behaviour change promoters in their communities. However, government-run CHW programmes have come and gone, with several programmes initiated and then cancelled or left to languish due to changing priorities and cost concerns (e.g. Community Health Volunteers, later called Village Health Guides, in the 1980s) [34, 63]. Launched in 2005, the ASHA programme is the latest in this line of CHW programmes. ASHAs are female community members with at least 8 years education (e.g. have completed elementary school), who receive 23 days initial training and perform five key activities: home visits, community meetings, monthly meetings at the primary health centres, facilitating outreach services within the village, and maintaining records [16]. ASHAs are now in place across rural India at a ratio of one per 1000 population and are increasingly also selected in marginalized urban settlements.	

Gender analysis in human resources for health highlights the overrepresentation of women as unpaid and often unrecognized carers for sick or elderly family members [4]. Within the paid health workforce, documented inequalities between male and female health workers in terms of pay, promotions, and employment security, and the higher rates workplace violence, including sexual violence, faced by female health workers remain pervasive [5–7]. With regard to CHWs in particular, studies have found that women generally prefer that CHWs who deliver reproductive, maternal, and child health interventions be female, because norms limit interaction between women and men who are not members of the same family. However, female CHWs are themselves limited by social norms constraining movement beyond the home and are vulnerable to critique and censure if they are seen to violate norms, such as by travelling outside the home at night or speaking to men [8]. Apart from the cross-cutting influence of social norms, gender dynamics influence CHWs at individual (family and intra-household relationships), community (safety and mobility), and health systems (remuneration and career progression) levels [9].

While the gender dimensions above refer to the technical content of CHW programmes, whether these dimensions are considered and how they are addressed enables one to categorize the extent to which a programme is gender responsive. Different terminologies exist ranging from gender blind, exploitative, accommodating, sensitive, specific, and transformative [10, 11]. Programmes can be considered gender blind if they ignore gender dimensions entirely, gender exploitative if they reinforce or take advantage of gender inequalities and stereotypes, gender accommodating if they address certain needs without changing the underlying inequalities that frame those needs, or gender transformative if they not only address needs, but transform the underlying power relations that maintain gender inequality [11].

Our analysis, conducted by internal programme leaders and external academic partners, takes into consideration published research, policy documents, and key informant interviews to explore the gendered design, evolution, and ongoing adaptations of the ASHA programme. In doing so, our objective is to reflect on the experience and challenges of addressing gender in large-scale CHW programmes that are a key but underappreciated foundation of health systems worldwide.

## Methods

Data sources for this analysis consisted of all publically available government policy documents on the ASHA programme, all published academic literature on ASHAs, and key informant interviews with both governmental and non-government actors at the national and state levels. These key informant interviews served to triangulate the embedded and tacit knowledge of this paper’s government-affiliated co-authors (RV, GG, SS, AS), who have worked with the Indian Ministry of Health and Family Welfare on the frontlines of developing and supporting the ASHA programme since its launch in 2005.

### Government policy documents

We identified all national level government policy documents in the public sphere on the ASHA programme (*n* = 96) through targeted searches of relevant Government of India webpages. These documents included federal government memos to state governments, parliamentary committee reports, training guidelines, meeting minutes, and policy updates. All documents were downloaded in full and underwent data extraction. Appendix 1 presents the full list of included policy documents.

### Academic literature review

**Fig. 1:**
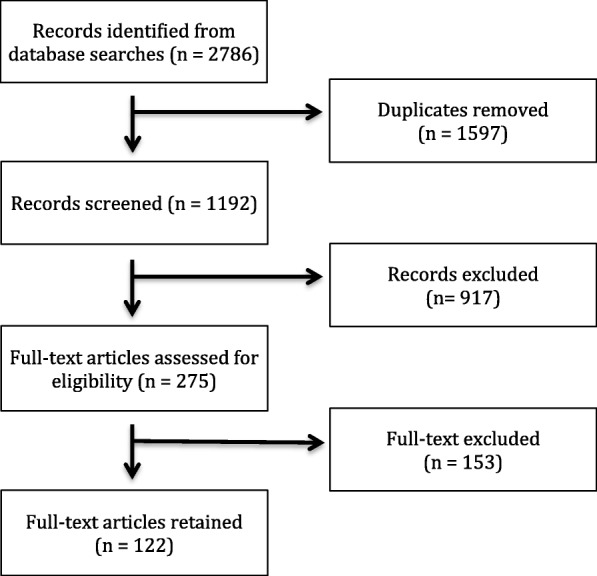
Diagram of academic article selection process

We searched the electronic databases PubMed, Embase, and Scopus for articles published between 1 January 2005 and 15 August 2016. Searches were developed in consultation with an academic librarian and incorporated keywords and free text for the concepts ASHA (e.g. “accredited social health activist”, “community health worker”, “lay health worker”) under one string and India (e.g. India, “Arunachal Pradesh”, Assam, Bihar) under another string, with the two strings joined by the Boolean operator “AND”. See Appendix 2 for the full search strategy. Articles were excluded if it was not clear whether the CHW programme being discussed was the Government of India’s ASHA programme or if the article mentioned ASHAs only in passing. All primary research articles, abstracts, and commentaries on the ASHA programme were included (*n* = 122) and passed on to the data extraction phase (see the “Analysis” section) (Fig. 1).

### Key informant interviews

We conducted 12 key informant (KI) interviews with Indian health system actors who had extensive involvement in shaping, designing, implementing, and adapting the ASHA programme. Key informants were senior policymakers in national- and state-level government and civil society actors. Interviews lasted an average of 1.5 h and were conducted face-to-face or over the telephone according to the convenience of the respondent. Respondents were invited to reflect upon the following areas: (a) stakeholder engagement in the ASHA policy process, including differing perspectives and agendas; (b) negotiations and tensions on policy content including ASHA roles and remit, incentives, selection criteria and process, training, and community orientation; (c) contextual influences; (d) threats and challenges that the programme has dealt with and speculation on potential future challenges; and (e) innovations and successes that can inform other large scale CHW programmes and future Indian policy processes.

### Analysis

Analysis began with data extraction from the research articles and government policy documents using a framework of CHW-health system interfaces (CHW social profile and agency; CHW programme inputs; CHW-community interface; health services context; programme governance; programme outcomes; and programme impact). We then identified the health system components of the ASHA programme that emerged from the data as most pertinent to gender (remuneration, career progression/training, community recognition and relations, gender-based violence and safety) and triangulated them with key interviews. Finally, we mapped policy changes, academic findings, and stakeholder narratives together according to topic to identify and understand reasons for the programme’s features and their adaptation over time.

## Results

We trace the evolution of the ASHA programme from its instrumentalist origins that focused on ASHAs as a tool to enable the health system meet its goals, to increasing attention to the empowering potential of the programme despite the challenges faced by the ASHAs. We then discuss the programme’s ongoing adaptations, as it strives to better meet ASHA needs for economic security, personal growth, and safety.

### Instrumentalist origins

The National Rural Health Mission (NRHM) (and later National Health Mission (NHM)), of which the ASHA programme was a cornerstone, focused particularly on maternal, newborn, and infant mortality, which were also receiving global attention through Millennium Development Goal 4 and 5 [12, 13] and population stabilization, which was of interest to government and external donors [14]: “First, in NRHM phase 1… we had Millennium Development Goals, so we also needed to reach there” (KI_02); “The donors… wanted to use her for population stabilisation and the link worker model, they wanted to push that” (KI_01). The government’s focus on addressing pressing reproductive, maternal, and child health needs was reflected in a number of key policy decisions for the ASHA programme (Table 2**).**

**Table 2:** Early instrumentalist considerations: how can the ASHA enable the health system to achieve its goals?

Policy decision	Rationale
All-female cadre [16, 64]	Women best suited to address maternal and child health (KI_04)
Volunteer cadre [16, 64]	Partially remunerated through performance based incentives to ensure high levels of activity and enable programmatic flexibility (KI_02, KI_07, KI_09)
Selected locally [16, 64]	Close to and likely to remain in community [16]
Training close to villages [16, 64]	Reduce travel to increase ASHA attendance of trainings (KI_01)

Forming an all-female cadre of CHWs was considered appropriate because maternal and child health were female spheres and women had high unmet reproductive health needs. Developing a cadre of female CHWs met women’s reproductive and maternal health needs in a culturally appropriate manner by having women speak to other women about these issues. At the same time, choosing only to have female CHWs worked within existing gendered norms of caregiving to address child health rather than challenging patriarchal norms that framed women as being primarily responsible for child care. In the following quotation, a policymaker (KI_04) justifies building an all-female cadre, saying that women are better at engaging other women and at influencing child health-related practices. Furthermore, she argues that women are less likely to shift into informal and unlicensed medical practice compared to men, suggesting that women are better at resisting corruption or falling into malpractice:

From the family and community perspective, [having] women [CHWs] has really helped in several ways. One is that they are able to much better reach out to women and children as opposed to men. The second is that they are prone to much less corruption than men…. Women relate much better to interact with women and children and also are less prone to doing private practice. One of the problems earlier [with a prior male CHW program] was that every male would [want] to become a [unlicensed] doctor and then would start practicing. (KI_04)

The decision that ASHAs would be volunteers who would earn small incentives for performing specific tasks was grounded in the government’s considerations of how best to meet population health needs in an affordable and sustainable manner appropriate for the federal governance structure in India. Some members of civil society were deeply concerned that the NHM did not create salaried government positions for ASHAs, and suggested that the voluntary nature of the position was only considered appropriate because ASHAs are women: “I would like to see nine lakh [900,000 – the approximate number of ASHAs at the time] men being treated like this” (KI_09).

However, policymakers cited a number of reasons why government actors argued that a salaried position was not feasible. First, they noted that administrative and monitoring structures in the rural health system were already strained and were struggling to ensure the selection, retention and performance of existing frontline health workers. They were concerned that ASHAs would underperform if guaranteed a salary, as was an ongoing challenge with child nutrition workers (called anganwadi workers) and frontline nurse-midwives.

So they [other policy actors] have a very strong opinion that if we change it, her performance will go down, she will become like another anganwadi worker. She’ll stop working. So that is also one of the arguments that comes across at all levels. So even some of the state nodal officers don’t want it, they say its fine, have performance based work. Increase the amount of incentives for each task that she does, make sure that she gets good amount of money. But they are not willing to change it to fixed paycheck because they are very worried that she will become another, like another anganwadi worker. And then who will monitor her? Because she is sitting somewhere in the village, and on a regular basis you can’t monitor her. (KI_07)

A lot of work gets done because … incentives make the person work (KI_02)

The person there knows that she will have to work. If she will not work she will not get paid (KI_06)

Second, national government actors in the Ministry of Finance were resistant to creating a large new cadre of government employees, who would be entitled to lifelong employment and pensions. The NHM was always supposed to be a mission, which would eventually strengthen state-level capacity to manage health in a decentralized manner:

It [NHM] was just envisaged as a temporary mission and we expected the states to be strengthened and to take ownership and then go forward, because I mean, it is a state subject and the responsibility is actually of the states for health. (KI_02)

Given India’s federal structure, the national government felt it was important that it be left to the states to decide on financing mechanisms themselves for the long term.

Third, some government actors noted that the flexibility built into the ASHA programme would be compromised by a salaried government position, since this would necessitate the application of rigid bureaucratic standards for eligibility, selection, leave, and ongoing employment. As a voluntary position, the educational requirement could be relaxed in highly marginalized areas and new women could be recruited to replace inactive ASHAs more easily. Finally, some civil society actors and government policymakers agreed that the intended community orientation of ASHAs could be compromised by a salaried employee model.

A genuine woman who wants to do something for the community will lose out, she will lose out on that opportunity. Because once you make it a government thing [a salaried formal position], then you will have… that exam, that process and application…. It will actually convert you into government. That lady would just say I am not kind of interested. So that woman who today, through that simple process become an ASHA, and also makes her name in the community… maybe tomorrow she will not be able to access that. (KI_06)

Very consciously, the institution of ASHA was seen as a community institution rather than a paid employee of the government. She was never intended to be the last member of the government health bureaucracy. It was on this account that a conscious decision was taken to provide only performance based payments to ASHAs. [15]

An implicit assumption underlying this latter rationalization is that a community orientation would inherently serve marginalized groups rather than be captured by community elites. Given the intersectional nature of ASHA profiles and the political economy of local communities, this assumption may not have always been valid.

Other key policy decisions that were taken to further community and health system goals included selecting local women and holding trainings close to the villages. ASHAs were to be married women, since women move to their husband’s villages upon marriage—unmarried women would likely leave their natal village upon marriage and were thus not preferred for ASHA selection [16]. Training close to the villages was also developed to increase the likelihood that ASHAs would attend the trainings.

### Transformative potential and gendered challenges

Despite these instrumentalist origins, designing the programme for women and having it implemented by a female CHW cadre also created opportunities to empower women by elevating them in rural societies as role models and female leaders who spoke out about women’s rights [17–19]. Research studies document that becoming an ASHA enabled rural women to gain knowledge, status, and exposure beyond the village, as well as to access remuneration, albeit a limited amount [18–22]. ASHAs have reported an increased sense of empowerment and personal growth, in part through their belief in the social value of their work [18–21, 23–27]. Furthermore, ASHAs have worked to further women’s interests, particularly in Chhattisgarh state where Mitanins (the name for ASHAs there) have mobilized protests against alcoholism [23], supported women’s collectives and taken action against gender based violence [24].

Despite benefits derived from their work, ASHAs continue to face significant gendered challenges. Research findings have extensively documented ASHA worker dissatisfaction with their pay in relation to their workload and contribution and identified negative consequences linked to the limited remuneration structure [18, 20, 22, 26, 28–33]. Limitations on their movement outside the home and heavy domestic responsibilities often curtail ASHAs’ ability to perform their professional role [22, 28], and ASHA were discouraged from working and belittled by family members when their remuneration was delayed or when incentives were found to be extremely low [33]. Limited space for career progression is linked to low institutional recognition, demotivation, and curtailed opportunities for growth [19, 31, 34, 35]. ASHAs face sexual harassment by other health workers and community members, linked to their mobility and public profile [36]. In 2016, an ASHA was gang raped by community members and subsequently died, highlighting the extent of gender based violence and security concerns facing ASHAs [37].

In response to these issues, government health policy is engaged in ongoing efforts to improve ASHA wellbeing through increasing ASHA economic security, developing career progression strategies, and addressing gender based violence, as detailed below and in Table 3. While these policy changes are developed at the national level—with state level consultation—implementation varies widely according to state priorities.

**Table 3:** Increased focus on ASHA empowerment: how can the health system improve ASHA wellbeing?

Policy decision	Rationale
Increased economic incentives [65]	Meet ASHA need for income; encourage uptake of banking services among ASHAs (KI_01)
Social security measures [65–67]	Provide support to ASHA and her family in the form of life and accident insurance and pension [66, 67]
Residential training workshops [67]	Immersive learning, develop empowering knowledge and skills, build solidarity among ASHAs (KI_01)
ASHA as member secretary of village health committee [67]	ASHA moves beyond all-female, maternal health sphere, gains community importance as joint signatory to access the village untied fund of Rs. 10 000 (KI_07)
Government scholarships for higher education [65]	Provide career progression opportunities to meet rising ASHA aspirations (KI_01, KI_07)
Health facilities to provide rest accommodation [67]	Increase ASHA safety and comfort when escorting women to facilities (KI_01) [67]

### Ongoing and unanticipated policy adaptations

#### Economic security

Policymakers have engaged creative strategies to meet ASHA calls for improved economic security despite the barriers to creating salaried positions for ASHAs and the Ministry of Finance’s resistance to fixed salaries [38] .Over time, the number of remunerated tasks and the amount of remuneration for each task steadily increased, from six remunerated tasks in 2005 to 38 in 2017 [39]. Social security measures were also introduced for ASHAs to access pensions and enrol in health insurance programmes [40, 41]. ASHA payments were shifted from cash to bank transfers, which reduced scope for skimming by higher level functionaries and brought ASHAs into the modern banking system [41]. In addition, the NHM identified a set of recurring monthly activities that ASHAs could perform to enable predictable remuneration that in some ways simulated a salary [42].

The impetus for these improvements can be traced to several sources. Some stakeholders expressed a prevailing sense that insufficient government expenditure on the programme was the ultimate impediment to ASHA empowerment: “We need to spend more money if we want to really empower them” (KI_03). In other key informant interviews, policy actors expressed their personal commitment to better meeting ASHA needs as a rights issue and in recognition for the increasingly important role that ASHAs have taken on in the Indian health system. For example:

Everyone I think, deep down, feels guilty that ASHAs need to be sort of compensated in a better way… everybody feels that they must do something. (KI_02)

Civil society activists and ASHAs themselves, with coordination through labour unions, have engaged in ongoing agitation and strikes for improved pay (KI_02, KI_06, KI_07, KI_09). The extent of political mobilization varies by state context. Others have noted that labour strikes have occurred in states where support structures had failed ASHAs:

Such protests are happening more in the states where the ASHA’s support structures are weak. You know where she thinks that nobody is really taking care of her, there is no mentor, ASHA facilitators are either not there or not doing the job that they should be doing. The system ownership is not there. This is more in states like [state 1], [state 2], where the support structures have been very weak. So in those states we see this happening a lot. I have not heard of a strike in [state 3] and [state 4]. The ASHAs get really small amounts of money but the investment in the program by the state is so much that, you know, that ASHAs feel part of, ASHAs feel taken care of. But in states like [state 5] where nothing else works for the ASHAs, even the trainings don’t happen for years, these kind of strikes are bound to happen. (KI_07).

#### Career aspirations and personal growth

Since 2012, government policymakers have added a number of features to the programme to better meet ASHA career aspirations and offer scope for greater personal growth. Residential training workshops with crèche facilities have been introduced so that ASHAs can experience immersive learning, develop empowering knowledge and skills, and build solidarity with other ASHAs [43]. The increasing scope of work and corresponding additional training is vital for addressing ASHA aspirations, including the acquisition of new personal and health-related skills [34]. In an effort to meet ASHA aspirations for career progression, the NHM has committed to paying tuition for all ASHAs seeking to complete secondary school education through the Open School System and to give ASHAs preferred admission to nursing and midwifery schools [44].

#### Community recognition and relations

Government policy has positioned ASHAs as member secretary of village health committees, thereby creating leadership opportunities in the village beyond the feminized-sphere of reproductive, maternal, and child health [45] (KI_01). While rigid gender norms make it difficult for many ASHAs to step into these roles, some communities have increasingly accepted the ASHA as meeting convener, public speaker, and activist on issues beyond reproductive, maternal and child health [24, 45]. This normative shift can be attributed to community recognition that the ASHA is following the government’s mandate as well as to training and support that has increased ASHA skills and confidence [45].

#### Safety and gender-based violence

Gender-based violence has been increasingly discussed by the national government as both an issue ASHAs can help address in their communities and as a threat to ASHAs themselves as they carry out their work [37, 46], particularly for lower caste ASHAs or ASHAs who transgress caste and other social hierarchies. Some ASHAs have begun taking action to mobilize their peers to reduce gender based violence [17]. The NHM sought to further support these efforts by developing a training module on the same topic [47]. ASHA safety has increasingly been prioritized with requirements that health facilities create safe overnight rest rooms for ASHAs who have accompanied patients, monthly staff meetings include discussion on sexual harassment, higher level staff take action in cases where threats or harassment are reported, all staff undergo training on violence against women, and grievance redressal systems be strengthened [46]. While these policy guidelines are issued at national level, it is up to states to implement them and for local managers and staff to adhere to them. They are important supportive steps, but they do not by themselves automatically change entrenched gender norms.

## Discussion

The ASHA programme is now over 10 years old and just under one million women strong, placing it among the largest health worker cadres in the world. Similar to many CHW programmes, it started with instrumentalist concerns: deploying poor women in rural areas on a volunteer basis to address family planning, maternal and child health concerns. Measures to ensure that she was married locally and that training took place close to villages accommodated gender norms rather than challenged them. Over time, feedback and demands from multiple stakeholders, both within and outside the programme’s formal support structures, have led to efforts to address their marginalized status. Key efforts include improving their remuneration and benefits within fiscal and administrative constraints, supporting their ongoing training and career pathways, bolstering their community recognition and leadership potential, and most recently responding to security concerns and gender-based violence.

The value of community embeddedness for CHW programmes is widely recognized as a mechanism to ensure programme relevance to local needs and secure community ownership, support, and recognition of CHWs [48, 49]. CHW programmes seek to align CHWs with communities and many do so in part by recognizing that cultural norms make female CHWs more acceptable to reaching female beneficiaries [50, 51]. However, such recognition rarely problematizes power structures within communities, including the intersection of caste, marital status, class or age with gender [9]. Although ASHA demographics show adequate representation of all castes [17, 52], becoming an ASHA does not transform caste-based aspects of women’s identity. Lower caste ASHAs were often unable to visit higher caste homes and sometimes experienced discrimination from other health workers [31], and higher caste ASHAs avoided and at times disparaged lower caste areas [19, 28, 53–55] and these patterns of caste hierarchy among women are replicated across South Asia [19, 26, 56]. Older female CHWs may not be trusted by adolescents, while unmarried CHWs may not be accepted by older mothers [50]. More fundamentally, a gender transformative approach would seek to engage both men and women in changing conservative gender norms, rather than reaffirm conformity to them [57]. Ongoing research that critically engages with community power dynamics will enable CHW policy to maximize the benefits of community embeddedness while recognizing and responding to CHWs’ capacity to challenge or promote problematic norms related to caste, class or age.

The issue of remunerating female CHWs is also nuanced. The assumption that poor women can set aside time for volunteer work is deeply problematic [58], as are the structural barriers that often prevent their progression in the health workforce [9]. At the same time, this does not negate the fact that female CHWs, including ASHAs, are empowered by the exposure and social affirmation gained through their work, even if it entails juggling domestic responsibilities, livelihood demands and volunteer work [59, 60]. The former however does not preclude measures to support household recognition and sharing of domestic responsibilities and more secure economic security for women. The ASHA programme demonstrates the complexities involved in improving ASHA remuneration and benefits at scale in a federal health system.

Both Pakistan and Ethiopia’s CHW programmes, while being instrumentalist in nature, were also established through political decisions that framed the initiatives as employing and empowering women in rural areas. Despite the political backing behind these programmes and the ASHA programme, the extent and nature of that empowerment remains an open question. While gender theories put forward a linear view of moving from gender blindness to transformation, the ASHA programme demonstrates that change is not linear nor always anticipated. While some policy actors were conscious of how problematic the initial framing of the ASHA programme was, it was not until later that evidence and calls for reform enabled them put in place systems to respond. Finally, while policy guidelines supporting gender equality can be passed nationally, they do not result in transformation if they are not owned and championed by local actors.

Particularly telling is the scepticism by some stakeholders about the ASHAs’ political mobilization and demands for higher pay, revealing gendered assumptions that female CHWs ought to display an inherently altruistic, acquiescent, and apolitical nature. The ASHAs’ political mobilization has gone against the narrative of selfless women, invested in the wellbeing of children and their communities at the expense of themselves. ASHAs have demonstrated agency in seeking more income, as well as courageously standing up for women’s rights in the face of pervasively conservative gender norms. Their agency to voice their demands illustrates that, regardless of whether a programme is instrumentalist or transformative in nature, more powerful actors cannot predict what female CHWs will do as increasingly empowered agents of change.

## Conclusions

This analysis identified the gender trade offs and complexities inherent to developing and sustaining a CHW programme at scale within a challenging context of patriarchal norms, health system hierarchies, federal governance structures, and rapidly evolving aspirations, capacities, and demands from female CHWs and the communities in which they reside. While the ASHA programme has consciously tried to move past its instrumentalist origins, continual adaptations are required to address ongoing gender challenges, both anticipated and unanticipated. These reforms, while imperfect due to the complexities faced, do chart progress and highlight the investments and responsive planning required to support gender transformation in CHW programmes.

## Appendix 1

**Table 4:** Publically available government policy documents on the ASHA programme

	Author	Title	Date
1	Dr. Manoj Kumar, Assistant Commissioner to Government of India	Constitution of Mentoring Group for ASHA	05.07.2005
2	Ministry of Health and Family Welfare (MoHFW)	Janani Suraksha Yojana- Guidelines for Implementation	2005
3	National Rural Health Mission (NRHM), MoHFW	FAQ ASHA and NRHM	2005
4	NRHM, MoHFW	FAQs ASHA	2005
5	S. Jalaja, Additional Secretary & Mission Director (NRHM)	Financial Guidelines for ASHA Support System	06.10.2006
6	NRHM, MoHFW	Accredited Social Health Activist (ASHA) Guidelines	2006
7	NRHM, MoHFW	Guideline on ASHA	2006
8	MoHFW	First Common Review Mission	2007
9	MoHFW	Monthly Village Health Nutrition Day-Guidelines for AWWs/ASHAs/ANMs/PRIs	2007
10	G C Chaturvedi, Additional Secretary & Mission Director (NRHM)	Performance based payment to ASHAs	19.09.2008
11	Amarjeet Sinha, Joint Secretary	Setting up ASHA Mentoring Group at the State/District/Block Level	16.07.2008
12	S. Swain, P. Swain, K. S. Nair, N. Dhar, S. Gupta and D. Nandan	A rapid appraisal of functioning of ASHA under NRHM in Cuttack, Orissa	2008
13	N. Jain, N. K. Srivastva, A. M. Khan, N. Dhar, V. Adhish, S. Menon and D. Nandan	Assessment of the functioning of ASHAs under NRHM in Uttar Pradesh.	2008
14	B. Mohapatra, U. Datta, S. Gupta, V.K. Tiwari, K.S. Nair, V. Adhish and D. Nandan	An Assessment of functioning and impact of Janani Suraksha Yojana in Orissa	2008
15	H. Haider,V.Adhish,S. Gupta,N. Dhar,U. Datta, S. Menon and D. Nandan	A Rapid Appraisal of SAHIYYA (ASHA) in Jharkhand	2008
16	M. K. Mohanty, S. Das, M. M. Misro, P. Kumar, J. P. Shivadasani and D. Nandan	Rapid Appraisal of Functioning of Village Health and Sanitation Committees (VHSCs) under NRHM in Orissa	2008
17	MoHFW	Second Common Review Mission	2008
18	Jyotsana Varma Ray, Director (PF-II)	Payment of monthly honorarium @ Rs.500/- to ASHA in addition to Performance incentives available under various programmes	13.04.2009
19	Amarjeet Sinha, Joint Secretary	Incentive payment to ASHAs in time	20.11.2009
20	Amarjeet Sinha, Joint Secretary	Model Guideline to Streamline refilling of ASHA drug kit	17.11.2009
21	R. Bhatnagar, K. Singh, T. Bir, U. Datta, T.P. S. Raj and D. Nandan	Assessment of performance based incentive system for ASHA Sahyogine in Udaipur, Rajasthan	2009
22	MoHFW	Third Common Review Mission	2009
23	MoHFW	Operational Manual for implementation if Malaria Programme	2009
24	P.K. Pradhan, Additional Secretary and Mission Director (NRHM)	Module 6 & 7 for ASHAs- Guidelines for rolling out	09.08.2010
25	Amarjeet Sinha, Joint Secretary	Indicators for the ASHA Programme	04.01.2010
26	MoHFW	Fourth Common Review Mission	2010
27	MoHFW	Promotion of Menstrual Hygiene among Adolescent Girls (10–19) years in Rural areas- Operational Guidelines	2010
28	P.K. Pradhan, Special Secretary and Mission Director (NRHM)	Strengthening ASHA Resource Centers	15.03.2011
29	Dr. Ajay Khera, Deputy Commissioner, Child Health and Immunization	Introduction of Home Based New Born Care (HBNC) Scheme through ASHA	19.08.2011
30	Dr. Ajay Khera, Deputy Commissioner, Child Health and Immunization	Guidelines for implementation of IMNCI strategy and Module 6 & 7 training for ASHA	22.12.2011
31	NHSRC	Program Evaluation of the Janani Suraksha Yojana	2011
32	MoHFW	Fifth Common Review Mission	2011
33	MoHFW	HBNC Operational Guidelines	2011
34	MoHFW	Operational Guidelines on Facility Based Management of Children with Severe Acute Malnutrition	01.12.2011
35	NRHM, MoHFW	Handbook for ASHA facilitators	2011
36	NHSRC	ASHA Which way forward: Evaluation of the ASHA programme	2011
37	NHSRC	ASHA Which way forward: Executive Summary - Evaluation of the ASHA programme	2011
38	NHM, MoHFW	Framework for implementation: National Health Mission 2012–2017	2011
39	Anuradha Gupta, Additional Secretary and Mission Director, NRHM	Establishing a system for performance monitoring of the ASHA programme	03.04.2012
40	J.S. Mathur, Joint Secretary, Ministry of Drinking Water & Sanitation (MDWS) and Manoj Jhalani, Joint Secretary, MoHFW	Incentivized ASHA for promoting toilet usage (Joint letter Ministry of Drinking Water & Sanitation & MoHFW)	18.05.2012
41	Manoj Jhalani, Joint Secretary, Reproductive and Child Health	Revision of financial norms for implementing UIP-MSG	25.05.2012
42	Manoj Jhalani, Joint Secretary	Utilizing services of ASHAs for ensuring spacing in birth and incentivising her for the effort	31.05.2012
43	Manoj Jhalani, Joint Secretary (Policy)	Involve ASHA for facilitating VHSNC meetings	03.07.2012
44	Manoj Jhalani, Joint Secretary	Increasing educational qualification upto 10th standard for future ASHA selection	22.08.2012
45	Manoj Jhalani, Joint Secretary	Pension-Swavalamban Scheme	04.09.2012
46	MoHFW	Sixth Common Review Mission	2012
47	Dr. Suresh K. Mohammed, Director (Reproductive and Child Health)	Payment of Composite & uniform incentive to ASHAs under JSY-MSG Approval	06.02.2013
48	Dr. Manisha Malhotra, Dy. Commissioner (Maternal Health)	Enhancement of incentive to Primary Informer (ASHA, AWW etc) under the Maternal Death Review (MDR)	14.02.2013
49	Anuradha Gupta, Additional Secretary and Mission Director, NRHM	Co-opt one member from NAMG in the SAMG	16.04.2013
50	Manoj Jhalani, Joint Secretary	Career Opportunity for ASHA (Enrolling in Open schooling/auxiliary nurse midwife (ANM)/general nurse midwife (GNM) courses)	18.09.2013
51	Sujoy Mojumdar, Director (Water)	ASHA Incentive for motivating households to have tap connection.	2013
52	Limatula Yaden, Director (NHM)	Minutes of 1st Meeting of Mission Steering Group (MSG) of National Health Mission (NHM)	20.10.2013
53	MoHFW	Seventh Common Review Mission	2013
54	MoHFW	Enhancing Optimal Infant And Young Child feeding Practices	2013
55	MoHFW	Guidelines for Control of Iron Deficiency Anaemia (National Iron+ Initiative)	15.01.2013
56	MoHFW	Prevention of post partum haemorrhage (PPH) through Community Based distribution of Misoprostol- Operational Guidelines & Reference Manual	2013
57	MoHFW	Rashtriya Bal Swasthya Karyakram (RBSK)-Operational Guidelines	2013
58	MoHFW	Guidelines for Community Processes	26.06.2013
59	National Skill Development Corporation (NSDC)	Qualifications pack for Basic Health Volunteers (equivalent to ASHA)	2013
60	Anuradha Gupta, Additional Secretary and Mission Director, NRHM	Revision of existing incentives and Incentive to ASHAs for Routine & Recurring Activities -MSG Approval	03.01.2014
61	Anuradha Gupta, Additional Secretary and Mission Director, NRHM	Strengthening the Village and Sanitation Committees (VHSNC)	31.01.2014/ 03.02.2014
62	Anuradha Gupta, Additional Secretary and Mission Director, NRHM	Career Opportunity for ASHA (enrolling ASHAs in Open Schooling/ANM/GNM)	17.02.2014
63	Limatula Yaden, Director (NHM)	Corrigendum-Routine and Recurring ASHA Incentives	11.02.2014
64	Limatula Yaden, Director (NHM)	Letter cum guidelines for monthly meeting of ASHA at Primary Health Centres (PHCs) and Sub-Centres (SHC).	23.07.2014
65	Limatula Yaden, Director (NHM)	Certification of ASHAs and Accreditation of Associated Agencies in ASHA Training	15.01.2014
66	Dr. Rakesh Kumar, Joint Secretary	Enhanced Compensation Scheme-2014′ for sterilization services	20.10.2014
67	MoHFW	Eighth Common Review Mission	2014
68	MoHFW	HBNC Revised Operational Guidelines	2014
69	MoHFW	Child Death Review Operational Guidelines	2014
70	MoHFW	Rashtriya Kishor Swasthya Karyakram (RKSK) - Operational Framework	2014
71	MoHFW	Revised National Tuberculosis Control Program (RNTCP)—national guidelines for partnership	2014
72	MoHFW	Guidelines for ASHA & MAS in Urban context	17.01.2014
73	Manoj Jhalani, Joint Secretary	Letter cum guidelines for Accreditation of State/Dist. Training Sites & Certification of ASHA trainers	18.05.2015
74	Rakesh Kumar, Joint Secretary	Replaced Cotrimoxazole with Amoxicillin in ASHA HBNC kit	02.01.2015
75	Manoj Jhalani, Joint Secretary	Health & Wellness Centers Pilot under UHC to provide Comprehensive Primary Health Care	11.02.2015
76	C. K. Mishra, Additional Secretary and Mission Director, NHM	RBSK: Early identification of Birth Defects by ASHAs	22.06.2015
77	D. N. Sahoo, Under Secretary to The Government of India	Clarification on ASHA entitlements for completion of ANC under JSY	03.08.2015
78	K B Agarwal, Additional Secretary	Involvement of ASHAs for prevention and control of Acute Encephalopathy Syndrome (AES)	01.10.2015
79	Capt. Kapil Chaudary, Deputy Secretary (NHM)	Minutes of 2nd Meeting of Mission Steering Group (MSG) of National Health Mission (NHM)	26.03.2015
80	MoHFW	Ninth Common Review Mission	2015
81	MoHFW	Operational Guidelines on Kala-Azar (Visceral Leishmaniasis) Elimination in India	2015
82	NHSRC, MoHFW	Guideline for NGO Involvement	2015
83	NHSRC, MoHFW	Guidelines for Rogi Kalyan Samities in Public Health Facilities	2015
84	MoHFW	Report of Task Force on Comprehensive Primary Health Care Rollout	2015
85	Manoj Jhalani, Joint Secretary & Central Vigilance Comission (CVO)	Implementing training of ASHA Facilitators for improved RMNCH outcomes using PLA.	04.01.2016
86	Arun Kumar Panda, Additional Secretary	Mission Parivar Vikas-Family planning services	10.11.2016
87	Manoj Jhalani, Joint Secretary & CVO	DISHA (Digital Literacy training for ASHA)	09.09.2016
88	Manoj Jhalani, Joint Secretary	Heat Stroke Prevention and Management	16.05.2016
89	C K Mishra, Additional Secretary & Mission Director, NHM	Safety measures for ASHAs	29.01.2016
90	Dr. Ajay Khera, Deputy Commissioner & Incharge (Child Health)	Revised Guidance note for follow-up of low birth weight (LBW) and Special Newborn Care Unit (SNCU) discharged infants by ASHA	20.10.2016
91	Capt. Kapil Chaudary, Director (NHM)	Minutes of 3rd Meeting of Mission Steering Group (MSG) of National Health Mission (NHM)	17.05.2016
92	MoHFW	National Deworming Day Operational Guidelines	2016
93	MoHFW	Pradhan Mantri Surakshit Matritva Abhiyan (PMSMA)	27.06.2016
94	MoHFW	Management of Common Cancers-Operational Framework	26.08.2016
95	MoHFW	Prevention, Screening and Control of Common Non-Communicable Diseases - Operational Guidelines	21.06.2016
96	MoHFW	Mothers’ Absolute Affection (MAA): Programme for Promotion of Breast Feeding - Operational Guidelines	2016

## Appendix 2

### Literature search strategy

#### PubMed

Search concept #1: ASHA community health worker

}mitanin} OR}Mitanin} OR}sahyogini} OR}Sahyogini} OR}ASHA} or}Accredited Social Health Activist} OR}accredited social health activist} OR}health auxiliary} OR}frontline health workers} OR}frontline health worker} OR}outreach worker} OR}outreach workers} OR}lay health worker} OR}lay health workers} OR}village health worker} OR}village health workers} OR}volunteer health worker} OR}volunteer health workers} OR}voluntary health workers} OR}voluntary health worker} OR}community health agent} OR}community health agents} OR}health promoter} OR}health promoters} OR}Community Health Workers}[Mesh] OR}community health worker} OR}community health workers} OR}community health aide} OR}community health aides} OR}community health volunteer} OR}community health volunteers} OR}community health assistants} OR}community health assistant} OR}community health promoters} OR}community health promoters} OR}community volunteer} OR}community volunteers} OR}health extension workers} OR}health extension worker} OR}village health volunteer} OR}village health volunteers} OR}Community Health Nursing}[Mesh] OR}close-to-community provider} OR}close-to-community providers} OR}community-based practitioner} OR}community-based practitioners} OR}Community Practitioners} OR}Community Practitioner} OR}community-based practitioners} OR}community-based practitioner} OR}rural health auxiliaries} OR}Basic health worker} OR}Basic health workers} OR}Community health agent} OR}Community health agents} OR}Community health promoter} OR}Community health promoters} OR}Community health representative} OR}Community health representatives} OR}Community health volunteer} OR}Community health volunteers} OR}Community resource person} OR}Female multipurpose health worker} OR}Female multipurpose health worker} OR}Health promoter} OR}Health promoters} OR}Outreach educator} OR}Outreach educators} OR}Sevika} OR}Village health helper} OR}Community Case Management Workers} OR}Community Health Agent} OR}Community Health Agents} OR}Community Health Care Provider} OR}Community Health Care Providers} OR}Community HealthCare Provider} OR}Community HealthCare Providers} OR}Cxommunity Health Extension Worker} OR}Community Health Extension Workers} OR}Family Health Worker} OR}Family Health Workers} OR}Family Planning Agent} OR}Family Planning Agents} OR}Family Welfare Assistant} OR}Family Welfare Assistants} OR}Female Community Health Volunteer} OR}Female Community Health Volunteers} OR}Health Agent} OR}Health Agents} OR}Health Assistant} OR}Health Assistants} OR}Maternal and Child Health Worker} OR}Maternal and Child Health Workers} OR}Peer Educator} OR}Peer Educators}.

Search concept #2: India

India[mesh] OR Indian[tiab] OR}Andhra Pradesh} [tiab] OR}Arunachal Pradesh} [tiab] OR Assam[tiab] OR Bihar[tiab] OR Chhattisgarh[tiab] OR Goa[tiab] OR Gujarat[tiab] OR Haryana[tiab] OR}Himachal Pradesh} [tiab] OR Jammu[tiab] OR Kashmir[tiab] OR Jharkhand[tiab] OR Karnataka[tiab] OR Kerala[tiab] OR}Madhya Pradesh} [tiab] OR Maharashtra[tiab] OR Manipur[tiab] OR Meghalaya[tiab] OR Mizoram[tiab] OR Nagaland[tiab] OR Odisha[tiab] OR Orissa[tiab] OR Punjab[tiab] OR Rajasthan[tiab] OR Sikkim[tiab] OR}Tamil Nadu} [tiab] OR Telangana[tiab] OR Tripura[tiab] OR}Uttar Pradesh} [tiab] OR Uttarakhand[tiab] OR}West Bengal} [tiab] OR Andaman[tiab] OR Nicobar[tiab] OR Chandigarh[tiab] OR Dadra[tiab] OR}Nagar Haveli} [tiab] OR Daman[tiab] OR Diu[tiab] OR Lakshadweep[tiab] OR Delhi[tiab] OR Puducherry[tiab] OR Pondicherry[tiab].

#### EMBASE

Search concept #1: ASHA community health worker

}mitanin} OR}Mitanin} OR}sahyogini} OR}Sahyogini} OR}ASHA} or}Accredited Social Health Activist} OR}accredited social health activist} OR ‘health auxiliary’/exp. OR}health auxiliary} OR}frontline health workers} OR}frontline health worker} OR}outreach worker} OR}outreach workers} OR}lay health worker} OR}lay health workers} OR}village health worker} OR}village health workers} OR}volunteer health worker} OR}volunteer health workers} OR}voluntary health workers} OR}voluntary health worker} OR}community health agent} OR}community health agents} OR}health promoter} OR}health promoters} OR}Community Health Workers} OR}community health worker} OR}community health workers} OR}community health aide} OR}community health aides} OR}community health volunteer} OR}community health volunteers} OR}community health assistants} OR}community health assistant} OR}community health promoters} OR}community health promoters} OR}community volunteer} OR}community volunteers} OR}health extension workers} OR}health extension worker} OR}village health volunteer} OR}village health volunteers} OR}Community Health Nursing} OR}Community Health Nursing}/exp. OR}close-to-community provider} OR}close-to-community providers} OR}community-based practitioner} OR}community-based practitioners} OR}Community Practitioners} OR}Community Practitioner} OR}community-based practitioners} OR}community-based practitioner} OR}rural health auxiliaries} OR}Basic health worker} OR}Basic health workers} OR}Community health agent} OR}Community health agents} OR}Community health promoter} OR}Community health promoters} OR}Community health representative} OR}Community health representatives} OR}Community health volunteer} OR}Community health volunteers} OR}Community resource person} OR}Female multipurpose health worker} OR}Female multipurpose health worker} OR}Health promoter} OR}Health promoters} OR}Outreach educator} OR}Outreach educators} OR}Sevika} OR}Village health helper} OR}Community Case Management Workers} OR}Community Health Agent} OR}Community Health Agents} OR}Community Health Care Provider} OR}Community Health Care Providers} OR}Community HealthCare Provider} OR}Community HealthCare Providers} OR}Community Health Extension Worker} OR}Community Health Extension Workers} OR}Family Health Worker} OR}Family Health Workers} OR}Family Planning Agent} OR}Family Planning Agents} OR}Family Welfare Assistant} OR}Family Welfare Assistants} OR}Female Community Health Volunteer} OR}Female Community Health Volunteers} OR}Health Agent} OR}Health Agents} OR}Health Assistant} OR}Health Assistants} OR}Maternal and Child Health Worker} OR}Maternal and Child Health Workers} OR}Peer Educator} OR}Peer Educators}.

Search concept #2: India

India/exp. OR (India OR Indian OR}Andhra Pradesh} OR}Arunachal Pradesh} OR Assam OR Bihar OR Chhattisgarh OR Goa OR Gujarat OR Haryana OR}Himachal Pradesh} OR Jammu OR Kashmir OR Jharkhand OR Karnataka OR Kerala OR}Madhya Pradesh} OR Maharashtra OR Manipur OR Meghalaya OR Mizoram OR Nagaland OR Odisha OR Orissa OR Punjab OR Rajasthan OR Sikkim OR}Tamil Nadu} OR Telangana OR Tripura OR}Uttar Pradesh} OR Uttarakhand OR}West Bengal} OR Andaman OR Nicobar OR Chandigarh OR Dadra OR}Nagar Haveli} OR Daman OR Diu OR Lakshadweep OR Delhi OR Puducherry OR Pondicherry):ab,ti.

#### Scopus

Search concept #1: ASHA community health worker

{mitanin} OR {Mitanin} OR {sahyogini} OR {Sahyogini} OR {ASHA} or {Accredited Social Health Activist} OR {accredited social health activist} OR ‘health auxiliary’/exp. OR {health auxiliary} OR {frontline health workers} OR {frontline health worker} OR {outreach worker} OR {outreach workers} OR {lay health worker} OR {lay health workers} OR {village health worker} OR {village health workers} OR {volunteer health worker} OR {volunteer health workers} OR {voluntary health workers} OR {voluntary health worker} OR {community health agent} OR {community health agents} OR {health promoter} OR {health promoters} OR {Community Health Workers} OR {community health worker} OR {community health workers} OR {community health aide} OR {community health aides} OR {community health volunteer} OR {community health volunteers} OR {community health assistants} OR {community health assistant} OR {community health promoters} OR {community health promoters} OR {community volunteer} OR {community volunteers} OR {health extension workers} OR {health extension worker} OR {village health volunteer} OR {village health volunteers} OR {Community Health Nursing} OR {Community Health Nursing}/exp. OR {close-to-community provider} OR {close-to-community providers} OR {community-based practitioner} OR {community-based practitioners} OR {Community Practitioners} OR {Community Practitioner} OR {community-based practitioners} OR {community-based practitioner} OR {rural health auxiliaries} OR {Basic health worker} OR {Basic health workers} OR {Community health agent} OR {Community health agents} OR {Community health promoter} OR {Community health promoters} OR {Community health representative} OR {Community health representatives} OR {Community health volunteer} OR {Community health volunteers} OR {Community resource person} OR {Female multipurpose health worker} OR {Female multipurpose health worker} OR {Health promoter} OR {Health promoters} OR {Outreach educator} OR {Outreach educators} OR {Sevika} OR {Village health helper} OR {Community Case Management Workers} OR {Community Health Agent} OR {Community Health Agents} OR {Community Health Care Provider} OR {Community Health Care Providers} OR {Community HealthCare Provider} OR {Community HealthCare Providers} OR {Community Health Extension Worker} OR {Community Health Extension Workers} OR {Family Health Worker} OR {Family Health Workers} OR {Family Planning Agent} OR {Family Planning Agents} OR {Family Welfare Assistant} OR {Family Welfare Assistants} OR {Female Community Health Volunteer} OR {Female Community Health Volunteers} OR {Health Agent} OR {Health Agents} OR {Health Assistant} OR {Health Assistants} OR {Maternal and Child Health Worker} OR {Maternal and Child Health Workers} OR {Peer Educator} OR {Peer Educators}.

Search concept #2: India

TITLE-ABS({India} OR {Indian} OR {Andhra Pradesh} OR {Arunachal Pradesh} OR {Assam} OR {Bihar} OR {Chhattisgarh} OR {Goa} OR {Gujarat} OR {Haryana} OR {Himachal Pradesh} OR {Jammu} OR {Kashmir} OR {Jharkhand} OR {Karnataka} OR {Kerala} OR {Madhya Pradesh} OR {Maharashtra} OR {Manipur} OR {Meghalaya} OR {Mizoram} OR {Nagaland} OR {Odisha} OR {Orissa} OR {Punjab} OR {Rajasthan} OR {Sikkim} OR {Tamil Nadu} OR {Telangana} OR {Tripura} OR {Uttar Pradesh} OR {Uttarakhand} OR {West Bengal} OR {Andaman} OR {Nicobar} OR {Chandigarh} OR {Dadra} OR {Nagar Haveli} OR {Daman} OR {Diu} OR {Lakshadweep} OR {Delhi} OR {Puducherry} OR {Pondicherry}).

## Abbreviations

ASHA: Accredited social health activist
CHW: Community health worker
KI: Key informant
NHM: National Health Mission
NRHM: National Rural Health Mission
